# The CLEAR (Considering Leading Experts’ Antithrombotic Regimes around peripheral angioplasty) survey: an international perspective on antiplatelet and anticoagulant practice for peripheral arterial endovascular intervention

**DOI:** 10.1186/s42155-019-0079-8

**Published:** 2019-11-12

**Authors:** Kitty H. F. Wong, Dave C. Bosanquet, Graeme K. Ambler, Mahim I. Qureshi, Robert J. Hinchliffe, Christopher P. Twine, CLEAR survey collaborators, Aldo Betanco, Andrea Mingoli, Andrej Isaak, Andrew Holden, Andrew Tambyraja, Angeliki Argyriou, Anthony Dean Godfrey, Ashraf Hassouna, Athanasios Diamantopoulos, Athanasios Saratzis, Atif Sharif, Ayoola Awopetu, Brennig Gwilym, Calvin Eng, Carlo Maturi, Charutha Senaratne, Christopher Graham, Colin Oliver, Raphael Coscas, Cristina L. Espada, Eamon Kavanagh, Eckhard Klenk, Efthymios Beropoulis, Esau Martinez, Eustratia Mpaili, Fabio Verzini, Fernando Gallardo, Gabriele Piffaretti, Gianni Celoria, Gladiol, Gonzalo P. Tapia, Greta Saggu, Hannah Travers, James Gordon-Smith, James Kirk, James Olivier, Jason Chuen, Jennifer Buxton, Jiber Hamid, John Quarmby, Jonathan Nicholls, Konstantinos Stavroulakis, Laura Drudi, Marco V. Usai, Mariano Rotger, Michael Gawenda, Mihai Ionac, Muayyad Almuhdhafer, Ng Jun Jie, Nicola Troisi, Nikesh Dattani, Nikolaos Patelis, Paolo Sapienza, Pasqualino Sirignano, Pierfrancesco Lapolla, Raveen Nijjer, Rengarajan Rajagopal, Roberto Farraresi, Rodrigo Biagioni, Rohan Pancharatnam, Sandeep Bahia, Simona Sica, Staros Spiliopoulos, Stefano Fazzini, Tanya Moledina, Tasleem Akhtar, Thomas Aherne, Thomas Broszey, Tony Moloney

**Affiliations:** 1 Bristol Centre for Surgical Research, University of Bristol, Bristol, BS8 2PS UK; 2 North Bristol NHS Trust, Southmead Hospital, Southmead Road, Bristol, BS10 5NB UK

**Keywords:** Platelet Aggregation Inhibitors, Peripheral Arterial Disease, Endovascular Procedures, Surveys and Questionnaires

## Abstract

**Background:**

Antiplatelet and anticoagulant therapy are commonly used before, during and after peripheral arterial endovascular intervention. This survey aimed to establish antiplatelet and anticoagulant choice for peripheral arterial endovascular intervention in contemporary clinical practice.

**Methods:**

Pilot-tested questionnaire distributed via collaborative research networks.

**Results:**

One hundred and sixty-two complete responses were collected from responders in 22 countries, predominantly the UK (48%) and the rest of the European Union (44%). Antiplatelet monotherapy was the most common choice pre-procedurally (62%). In the UK, there was no difference between dual and single antiplatelet therapy use post procedure (50% vs. 37% *p* = 0.107). However, a significant majority of EU respondents used dual therapy (68% vs. 20% *p* < 0.001). There was variation in choice of antiplatelet therapy by the device used and the anatomical location of the intervention artery. The majority (82%) of respondents believed there was insufficient evidence to guide antithrombotic therapy after peripheral endovascular intervention and most (92%) would support a randomised trial.

**Conclusions:**

There is widespread variation in the use of antiplatelet therapy, especially post peripheral arterial endovascular intervention. Clinicians would support the development of a randomised trial comparing dual antiplatelet therapy with monotherapy.

## Background

Antiplatelet therapy is commonly used before, during and after peripheral arterial endovascular intervention. It is reccomended in guidelines; predominantly due to a reduction in cardiovascular events (Conte et al. 2019; National Institute for Health and Care Excellence 2015). However the clinical benefit to support the use of specific antiplatelet regimens, even for ‘established’ indications such as a reduction in cardiovascular events, is actually marginal (Ambler et al. 2019). As a result, guidelines are conflicted as they interpret the evidence differently (Conte et al. 2019; National Institute for Health and Care Excellence 2015). The evidence for maintenance of lower limb bypass patency (Ambler et al. 2019) and when used following coronary intervention (Levine et al. 2016) is more established. This is probably why randomised trials examining new technologies for use in the peripheral arteries have mandated the use of dual antiplatelet therapy in the intervention arms, but not always the comparator arm (Krankenberg et al. 2007). Perhaps as a result the use of dual antiplatelet therapy after peripheral endovascular intervention has become more prevalent in clinical practice. Add in the constantly emerging evidence in cardiology (Levine et al. 2016), and well publicised trials of direct oral anticoagulants for non-intervened peripheral arterial disease (Anand et al. 2018), and it has become even more confusing for peripheral endovascular practitioners. The benefits of any change in drug regimen or new drug have to be balanced against the risks they pose and the cost (Ambler et al. 2019). When compared to cardiology there is a huge gap in our knowledge for antiplatelet therapy choice around peripheral arterial endovascular intervention; a high-volume procedure which is becoming more common (Cull et al. 2010). The aim of this survey was to establish contemporary practice for antiplatelet and or antithrombotic therapy to inform a clinical trial design comparing antiplatelet or antithrombotic agents after peripheral arterial endovascular intervention.

## Methods

As no validated reporting guidelines for surveys exist, we followed the 38 point checklist generated from a systematic review of published guidance and reporting practice (Bennett et al. 2011). The survey was designed by the authors (KW, DCB, RJH, CPT) and piloted on 7 vascular surgeons and 2 vascular interventional radiologists at North Bristol NHS Trust. A final 9 item survey was defined (Additional file 1).

### Survey protocol, inclusion criteria and dissemination

The United Kingdom based trainee collaborative VERN (Vascular and Endovascular Research Network) agreed to disseminate the survey (Bosanquet et al. 2016). A predefined protocol was specified for the survey and made available on the VERN website (available at https://vascular-research.net/projects/considering-leading-experts-antithrombotic-regimes-after-peripheral-angioplasty-clear-survey/ and included as Additional file 1). This defined the surveyors as any grade in training and respondents as consultant (or equivalent level) vascular interventional radiologists and vascular surgeons working in a centre treating vascular patients. To ensure responses were from consultants and to eliminate duplication, the email addresses of the respondents were collected. To qualify for collaborative authorship surveyors had to collect and input 5 responses. The survey was distributed as an online SurveyMonkey form via the VERN emailing list. Social media was used for advertising. Vascupedia, the Rouleaux Club, Association of Surgeons in Training (ASIT), and STARSurg also distributed the survey via their emailing list. The survey was open from 22nd July to 29th August 2019. Data were coded, collected and stored anonymously on encrypted devices.

### Statistical analysis

Incomplete responses were removed during data cleaning. Five percent of the complete responses were randomly selected and independently verified by directly contacting the responder; correlation was 100%. Categorical data are presented as counts and percentages per group. Statistical comparisons were performed via a predetermined protocol. Significant differences between categorical variables were determined via Chi-squared tests in Excel (Microsoft, version 16.16.4). Results were further analysed and compared by dividing the sample into geographical subgroups if sufficient responses (> 50) were collected. Responses to questions about antithrombotic therapy for different arterial levels were modelled using a mixed effects logistic regression model with a random intercept and fixed slope using the ‘lme4’ package in the R statistical programming environment. The model compared a preference for dual antiplatelet therapy over monotherapy, with participants who expressed a preference for other options removed. All statistical tests were 2-sided with a 0.05 level of significance.

## Results

**Fig. 1:**
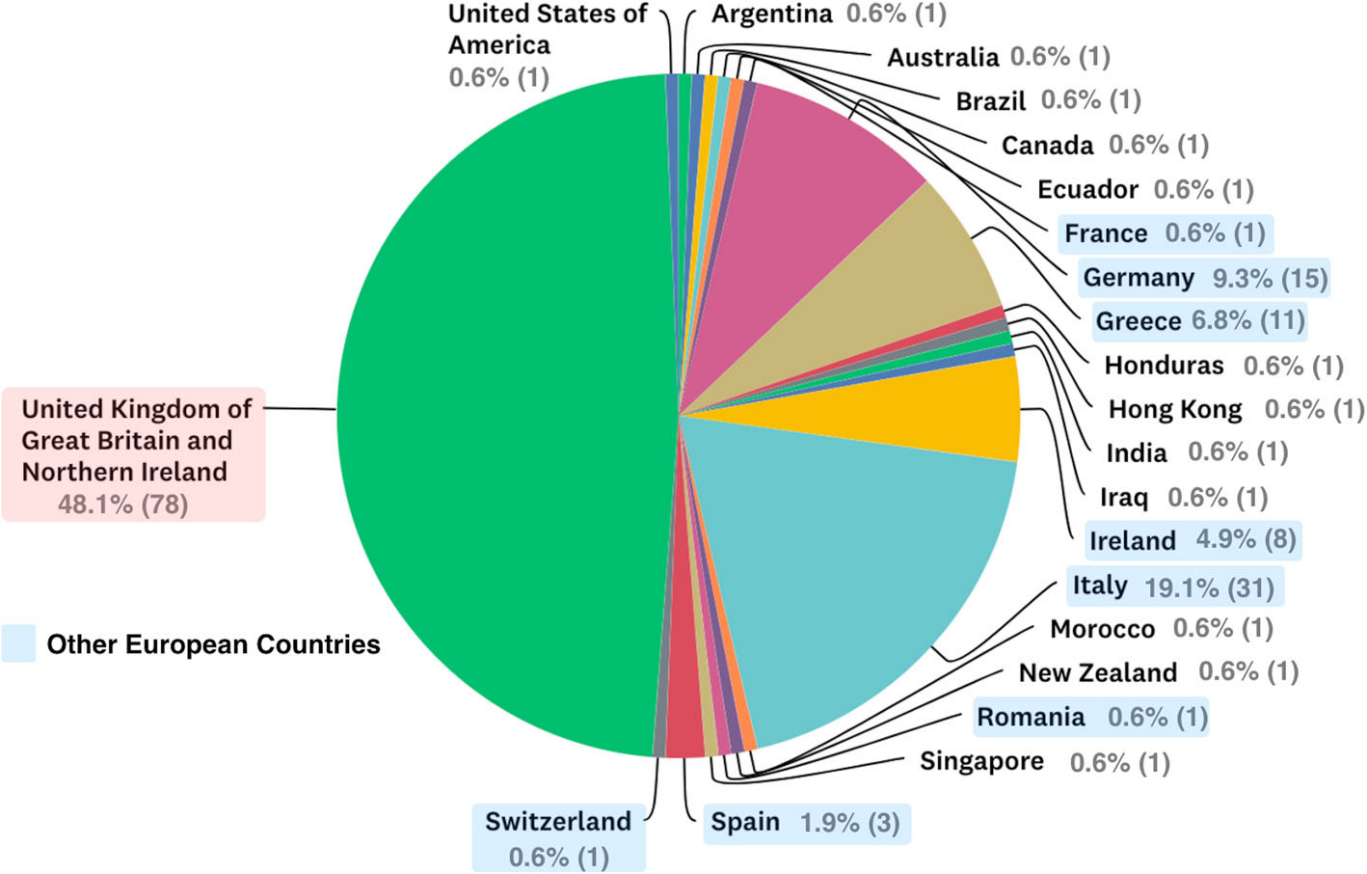
Respondents country of work

One hundred and eighty-three responses were received from discrete responders, obtained by 71 surveyors. Twenty-one were incomplete, leaving 162 complete responses. Of these, 78 (48%) were from the UK, 71 (44%) from other European Union (EU) countries and 13 (8%) from other countries (Fig. 1). The exact response rate to the questionnaire was impossible to calculate because of the way the survey was distributed using research networks and social media. It was also not possible to calculate the number of consultants and trainees included in the emailing lists as this information was not routinely captured.

### Pre-procedural antithrombotic therapy

**Fig. 2:**
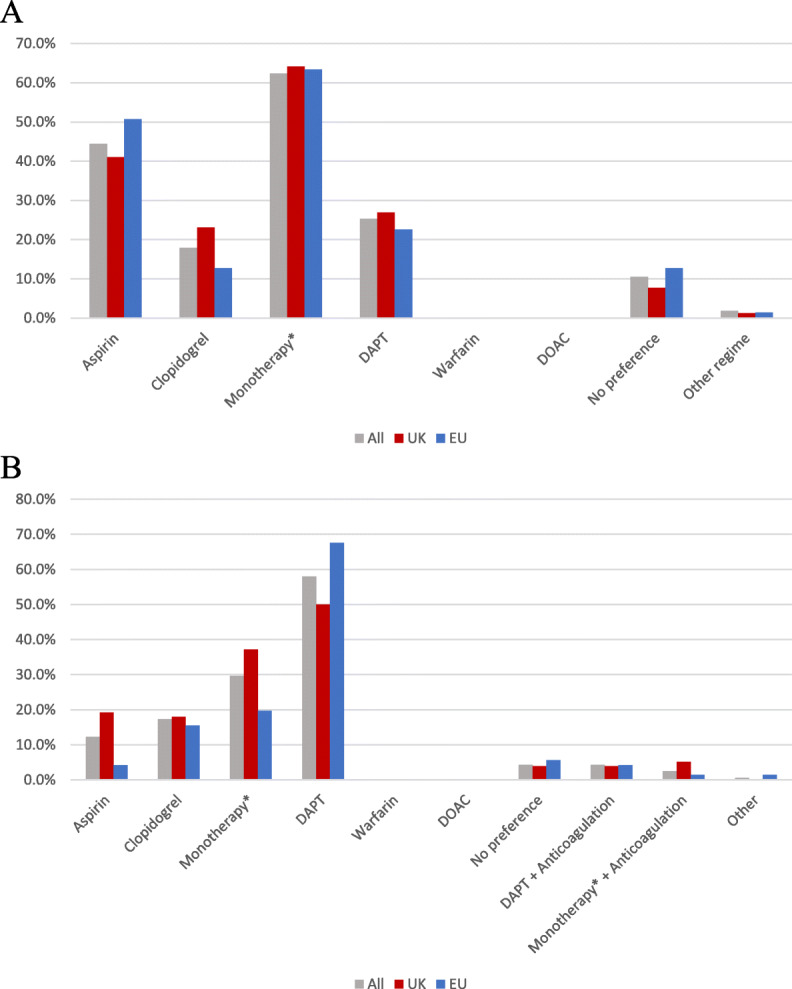
Respondent preference for pre (**a**) and post (**b**) procedural antithrombotic therapy. Legend: DAPT dual antiplatelet therapy. DOAC direct oral anticoagulant. *Monotherapy = composite of aspirin, clopidogrel and ‘other regime’ where an antiplatelet agent was specified

Antiplatelet monotherapy was the most popular choice for pre-procedural antithrombotic therapy from 62% of respondents (Fig. 2a). Aspirin was used most frequently (44%) while clopidogrel was preferred by 18%. The majority of respondents would continue aspirin or clopidogrel monotherapy for the procedure (98% and 80% respectively), while most would stop Warfarin and direct oral anticoagulants (94% and 95%). Fifty one percent would change from dual therapy to monotherapy; this was significantly higher in the UK group compared to the EU group (60% vs. 41%, *p* = 0.018). Most (99%) of respondents used a loading dose of unfractionated heparin intraprocedurally. Four (3%) would additionally give aspirin, clopidogrel or dual antiplatelet therapy.

### Post procedural antithrombotic therapy

Overall, more respondents would use dual antiplatelet therapy (58%) than monotherapy (30%) after an intervention (*p* < 0.001). Anticoagulants were not used in isolation by any responders but were used by 5% in conjunction with dual antiplatelet therapy. In the UK, there was no difference between dual and monotherapy use post procedure (50% vs. 37% *p* = 0.107). However, a significant majority of EU respondents used dual therapy (68% vs 20% *p* < 0.001).

### Antithrombotic differences by procedure and anatomical location

**Fig. 3:**
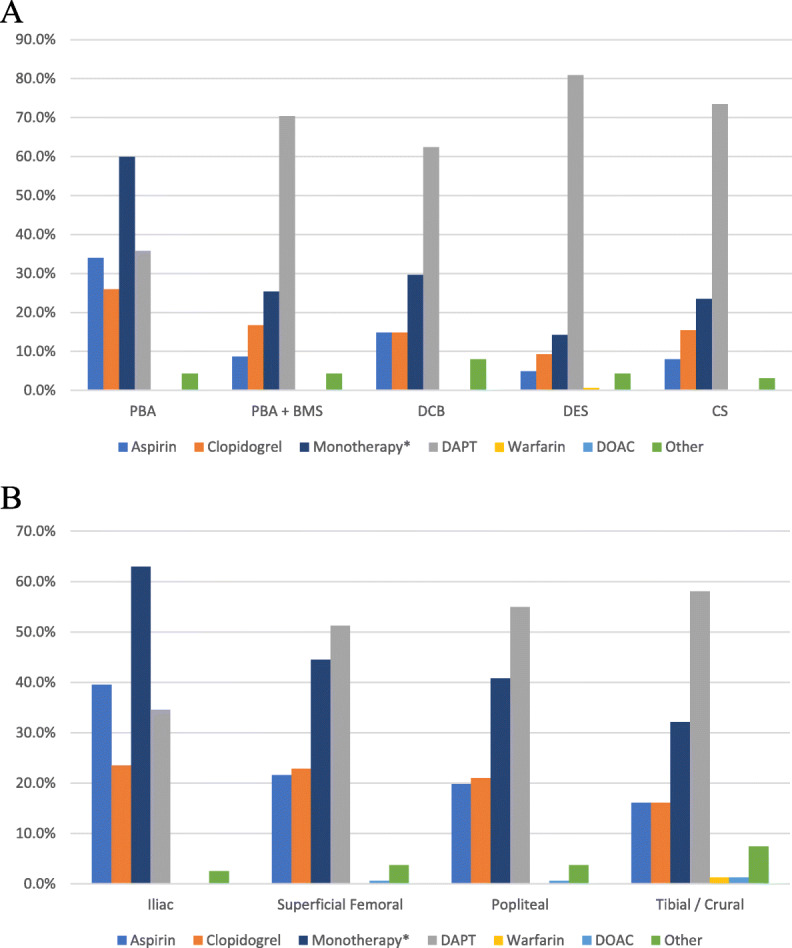
Antithrombotic therapy stratified by procedure (**a**) and anatomical location (**b**). Legend: DAPT dual antiplatelet therapy. DOAC direct oral anticoagulant. PBA Plain balloon angioplasty. BMS Bare metal stent. DCB Drug coated balloon. DES Drug eluting stent. CS Covered stent. *Monotherapy = composite of aspirin, clopidogrel and ‘other regime’ where an antiplatelet agent was specified

Antiplatelet monotherapy was more commonly preferred following plain balloon angioplasty, while dual therapy was more commonly preferred following every other type of endovascular treatment (Fig. 3a; *p* < 0.001 all comparisons). Interestingly, this was different to the preferences seen when asking generally about antithrombotic therapy post procedurally (Fig. 2b). Antiplatelet monotherapy was preferred by a higher proportion of respondents in the UK compared to the EU after drug-coated balloon angioplasty (36% vs 21%, *p* = 0.047). Monotherapy was preferred over dual antiplatelet therapy for iliac artery intervention (63% vs 35%, *p* < 0.001), while dual therapy was preferred for more distal lesions (Fig. 3b). Mixed-effects modelling revealed that this trend towards dual therapy was significant, with an odds ratio of 5.8 in favour of dual therapy per anatomical level of artery treated more distally (95% confidence interval 3.5–11.3, *p* < 0.001).

### Rationale for antithrombotic choice

**Fig. 4:**
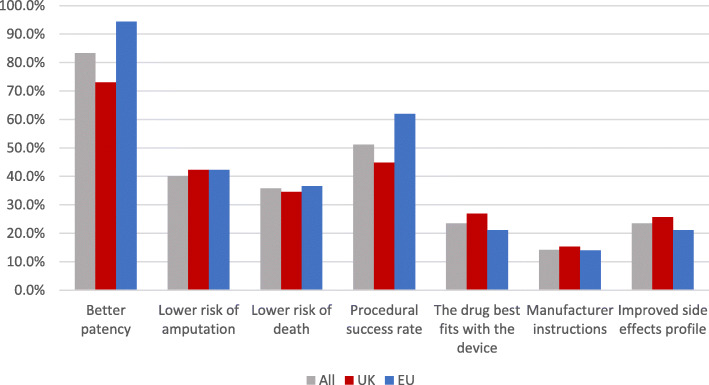
Rationale for antithrombotic choice

The majority (84%) of respondents chose antiplatelet therapy because of perceived improvements in patency rates (Fig. 4). The EU respondents chose this option significantly more frequently (94% vs. 73% *p* < 0.001). Procedural success was the next most frequent rationale (51%). Factors listed as ‘other’ were: Reduction in myocardial infarction (Conte et al. 2019); bleeding risk (National Institute for Health and Care Excellence 2015); patient preference (Conte et al. 2019); pre-operative condition (Conte et al. 2019); cost (Conte et al. 2019) and according to local expert advice (National Institute for Health and Care Excellence 2015). The majority (82%) of respondents believed there was insufficient evidence to guide antithrombotic therapy after peripheral endovascular intervention. This was higher in the UK than the EU (90% vs. 73% *p* = 0.009). The majority (92%) would be interested in participating in a randomised trial on this topic.

## Discussion

Internationally, dual antiplatelet therapy is now chosen more commonly than monotherapy after peripheral arterial endovascular intervention. UK based respondents used single and dual therapy equally, while the rest of the EU used dual therapy more commonly. Dual antiplatelet therapy is used significantly more commonly after more distal interventions. There was a difference in how responders chose antiplatelet regimens when asked about newer devices (such as drug coated or eluting devices), with dual therapy being chosen more frequently. This may have been influenced by the early company sponsored randomised trials (Krankenberg et al. 2007), which specified dual therapy when the devices were being used but not necessarily in the control arm. The instructions for use for several major devices also recommend post procedural dual antiplatelet therapy. Again, the reason is unclear. These choices may have contributed to the current clinical confusion and increased use of dual therapy after newer devices. Dual therapy was more commonly chosen as interventions became more distal, which is probably a reflection of the concern raised by responders about loss of patency. Patency is not necessarily related to limb loss, but it is the most commonly used surrogate outcome in randomised trials in the peripheral vasculature as it is easy to power a relatively small sample size, and it is easy to measure. As a result, responders tend to think in terms of patency (84% of responders) post intervention and not in terms of patient-centred outcomes. The widespread use of dual antiplatelet therapy is a concern because of the lack of evidence of benefit but clear evidence of harm (Ambler et al. 2019). For example, in all randomised trials comparing dual with mono antiplatelet therapy for peripheral arterial disease, dual antiplatelet therapy caused 37 more major bleeds per 1000 patients than monotherapy (*P* < 0.001) but did not clearly improve any post endovascular clinical outcome (Ambler et al. 2019). The Vascular community is behind cardiology in this respect, as there are a number of trials examining antiplatelet therapy after peripheral coronary intervention. While it is tempting to draw practice from these trials it is bad science to do so because of the differences between the flow dynamics in the coronary and peripheral arteries, and the differences in clinical sequelae of loss of patency in the heart and in the leg. The limitations of the survey include not stipulating a certain number of entries from a country to be included in the final analysis, non-response bias (which is a potential flaw in any survey or questionnaire), and response bias in that people who are using the antiplatelet regimes chosen may be more likely to be involved in the questionnaire. These biases should have been reduced by the study design, where surveyors chose respondents rather than respondents being self-selected. There will have been a proportionally higher number of responses from the UK because the distribution lists used were predominantly based in the UK. The other EU countries group was comprised of a low number of responses from a large number of countries. This means that while it is not a true ‘EU’ position the grouping is useful to highlight that there are differences in UK practice which would need to be taken into account for any randomised trial. There was a lack of information on doses of drugs in certain areas such as intraprocedural heparin. This was intentional to pragmatically keep the questionnaire relatively quick to complete but would have added interesting additional information.

## Conclusions

There is widespread variation in the use of antiplatelet therapy, especially following peripheral arterial endovascular intervention. Preferences differed by device used and anatomical site of procedure. The majority of interventional radiologists and surgeons agreed that there was insufficient evidence to make robust antiplatelet regime choices post endovascular intervention and would support a randomised trial.

## Supplementary information

**Additional file 1: Figure S1.** Published protocol and final survey.

**Additional file 2: Table S1.** Full data tables for responses.

## Data Availability

The full anonymised survey result dataset is included as a Additional file 2.

## Abbreviations

EU: European Union
UK: United Kingdom
VERN: Vascular and Endovascular Research Network
