# Correction to: The CLEAR (Considering Leading Experts’ Antithrombotic Regimes around peripheral angioplasty) survey: an international perspective on antiplatelet and anticoagulant practice for peripheral arterial endovascular intervention

**DOI:** 10.1186/s42155-020-00173-8

**Published:** 2020-11-27

**Authors:** Kitty H. F. Wong, Dave C. Bosanquet, Graeme K. Ambler, Mahim I. Qureshi, Robert J. Hinchliffe, Christopher P. Twine, CLEAR survey collaborators, Aldo Betanco, Andrea Mingoli, Andrej Isaak, Andrew Holden, Andrew Tambyraja, Angeliki Argyriou, Anthony Dean Godfrey, Ashraf Hassouna, Athanasios Diamantopoulos, Athanasios Saratzis, Atif Sharif, Ayoola Awopetu, Brennig Gwilym, Calvin Eng, Carlo Maturi, Charutha Senaratne, Christopher Graham, Colin Oliver, Raphael Coscas, Cristina L. Espada, Eamon Kavanagh, Eckhard Klenk, Efthymios Beropoulis, Esau Martinez, Eustratia Mpaili, Fabio Verzini, Fernando Gallardo, Gabriele Piffaretti, Gianni Celoria, Gladiol, Gonzalo P. Tapia, Greta Saggu, Hannah Travers, James Gordon-Smith, James Kirk, James Olivier, Jason Chuen, Jennifer Buxton, Jiber Hamid, John Quarmby, Jonathan Nicholls, Konstantinos Stavroulakis, Laura Drudi, Marco V. Usai, Mariano Rotger, Michael Gawenda, Mihai Ionac, Muayyad Almuhdhafer, Ng Jun Jie, Nicola Troisi, Nikesh Dattani, Nikolaos Patelis, Paolo Sapienza, Pasqualino Sirignano, Pierfrancesco Lapolla, Raveen Nijjer, Rengarajan Rajagopal, Roberto Farraresi, Rodrigo Biagioni, Rohan Pancharatnam, Sandeep Bahia, Simona Sica, Staros Spiliopoulos, Stefano Fazzini, Tanya Moledina, Tasleem Akhtar, Thomas Aherne, Thomas Broszey, Tony Moloney

**Affiliations:** 1 Bristol Centre for Surgical Research, University of Bristol, Bristol, BS8 2PS UK; 2 North Bristol NHS Trust, Southmead Hospital, Southmead Road, Bristol, BS10 5NB UK

**Correction to: CVIR Endovasc 2, 37 (2019)**

**https://doi.org/10.1186/s42155-019-0079-8**

Following the publication of the original article (Wong et al. 2019), the authors have discovered error in on one the collaborators. Coscas Raphael should be Raphael Coscas. As a result, this was not indexed properly.

The original article has been corrected.
