# Supplementary material for: The CLEAR (Considering Leading Experts’ Antithrombotic Regimes around peripheral angioplasty) survey: an international perspective on antiplatelet and anticoagulant practice for peripheral arterial endovascular intervention
Source: CVIR Endovasc. 2019 Nov 12;2:37. doi: 10.1186/s42155-019-0079-8 (PMC6966346; doi:10.1186/s42155-019-0079-8)
Supplement: Supplementary file 1 — Additional file 1: Figure S1. Published protocol and final survey. [file 42155_2019_79_MOESM1_ESM.pdf]

Vascular and Endovascular Research Network (VERN)

Considering **L**eading **E**xperts' **A**ntithrombotic **R**egimes after peripheral angioplasty (CLEAR) Survey

In collaboration with VERN.

With support from the Rouleaux Club.

Survey Protocol

21 July 2019

Table of Contents

Introduction **3**

Study Aims **3**

Methods **4**

Summary 4

Project Timeline 4

Centre Eligibility 4

Surveyor and Respondent Eligibility 4

Data Items 5

Data Collection 6

Authorship 6

Additional Information **6**

Writing Group **7**

About VERN **7**

References **9**

Appendix A: Survey Summary **10**

Introduction

Peripheral arterial disease (PAD) in the lower limbs may be treated using a combination of pharmacological and revascularisation techniques, such as open surgical bypass and percutaneous endovascular intervention. As PAD stems from a build-up of atherosclerotic plaques, antiplatelets and anticoagulants are a mainstay therapy to reduce cardiovascular risk. The National Institute for Health and Care Excellence (NICE) recommends antiplatelet therapy as secondary prevention for cardiovascular disease[1]. The American Heart Association / American College of Cardiology (AHA/ACC) guidelines also recommend antiplatelet therapy for patients with symptomatic PAD (class Ia)[2]. However, the guidance for antiplatelet therapy after endovascular intervention is less clear.

Antiplatelet agents have a major role in maintaining primary vessel patency post endovascular treatment and preventing restenosis. The rationale behind this is drawn from the fact that angioplasty and/or stenting can damage the arterial endothelium and initiate platelet aggregation, which can lead to unfavourable outcomes such as restenosis, stent failure, and thrombus formation[3]. The presence of cardiovascular risk factors can also affect patency rates[4]. Although the AHA/ACC guidelines suggests that dual antiplatelet therapy (aspirin and clopidogrel) may be reasonable after lower limb revascularisation (class IIb), there is no robust data to support such practice due to the lack of large, well-designed clinical trials[2]. As such, there is wide variation in practice in regards to the choice, duration, and dose of drug therapy without a strong and validated antiplatelet regimen[3].

Study Aims

- **Primary aim:** To assess interventional radiologist and vascular surgeons' current antiplatelet and anticoagulant prescribing practice after percutaneous endovascular intervention in the lower limb.
- **Secondary aims:**
    - To identify any patterns in antiplatelet and anticoagulant prescribing pre- and post-procedurally.
    - To explore the rationale behind different antiplatelet or anticoagulant protocols.
    - To explore equipoise around a randomised trial in this area.

Methods

Summary

Surveyors (any grade in training) will be expected to collect responses from consultant vascular surgeons or consultant interventional radiologists in regards to their antiplatelet and anticoagulation prescribing practice around percutaneous endovascular intervention in the lower limb. **Any surveyor collecting and inputting 5 responses will quality as a PubMed-citable collaborator under the "VERN Collaborators" on any publication based on the data**.

Project Timeline

- The suggested overall data collection will be from 22nd July to 19th August 2019 (4 weeks). This may be extended to give flexibility to include further UK or international centres.
- A report of the results will be produced by Thursday 5th September 2019. The results of the survey will be submitted for presentation and to an appropriate peer reviewed journal.

Centre Eligibility

- This survey is open to any hospital in the UK and Ireland that provides percutaneous endovascular interventions in the lower limb.
- International centres are also invited to participate.
- Local audit approval and registration is not required for this snapshot survey, as no patient data will be collected.

Surveyor and Respondent Eligibility

- **The person being surveyed (Respondent)** must be a consultant vascular surgeon or consultant interventional radiologist or equivalent grade.
- **The person completing the survey (Surveyor)** may be at any grade in their training, including medical students, foundation year doctors, core trainees, specialty trainees, and consultant vascular surgeons or interventional radiologists. Allied health professionals including vascular nurses and scientists are also invited to participate.
- **To qualify for collaborative authorship**, the collaborator must collect responses from at least 5 different consultants. Details regarding the collaborative authorship model can be found below in the *"Authorship"* section.

Data Items

A full summary of data fields is available below in *appendix 1*. The survey will collect data on surgeons' routine prescribing preference for antiplatelets and/or anticoagulant agents pre-, intra-, and post-endovascular intervention in the lower limb. **All questions refer to patients with no other indication for specific antiplatelet or anticoagulation aside from peripheral arterial disease.**

Definition of Terms:

- **Pre-procedural loading** is defined as any short-term loading dose of drug prior to lower limb endovascular intervention, including any 'slow loading' a few days before the procedure.
- **Post-procedural drug protocol** is defined as long-term drug prescription after peripheral endovascular interventions.
- **Antiplatelet and anticoagulant agents** include but are not limited to:
    - Aspirin 75mg
    - Clopidogrel 75mg
    - Dual antiplatelet therapy (aspirin and clopidogrel)
    - Other antiplatelet or dual regime (please specify)
    - Warfarin
    - Direct oral anticoagulants (DOAC) e.g. rivaroxaban, apixaban, dabigatran
- **Endovascular interventions** include:
    - Percutaneous transluminal angioplasty (PTA)
    - Bare metal stent (BMS)
    - Covered stent (CS)
    - Drug-eluting stent (DES)
    - Drug-coated balloon (DCB)
    - Atherectomy (AT)
- Antiplatelet and anticoagulant prescribing for open surgical procedures such as bypass surgery or endarterectomy are **not** relevant to this survey.

Data Collection

The survey will be distributed via the Vascular and Endovascular Research Network (VERN) as an online form. Survey Monkey will be used to collect and store responses. The details of the person completing the survey (email, name, hospital) will be stored for authorship purposes. The final results will be anonymised.

Data Validation

Incomplete responses will be removed as part of data cleaning. In order to ensure and safeguard collaborator contributions, a percentage of the interviews will be independently verified by directly contacting the interviewee to ensure responses are correct and valid.

Authorship

In accordance with the National Research Collaborative (NRC) authorship guidelines[5], the steering writing group will be listed as significant authors, followed by a single corporate authorship ("Vascular and Endovascular Research Network (VERN) Collaborators") under which all surveyors will be listed as PubMed-citable collaborators.

- **Writing Group:** A core group responsible for the overall protocol design, project co-ordination, data handling, data analysis, and preparation of research manuscripts.
- **Surveyors (Collaborators):** The person completing the survey over the specified period at a particular centre. To qualify as a VERN collaborator, the collaborator must collect responses from at least 5 different consultants.
- **Respondents:** Consultants who were surveyed but did not complete the survey themselves. *An acknowledgement will be made to consultants taking part in the study as a group, but no PubMed-citable collaborative authorship will be given.*

Additional Information

This survey is being undertaken as part of the planning for a randomised trial to compare different antithrombotic regimes after endovascular intervention for peripheral arterial disease. After completion, the survey report will be submitted for publication in a peer-reviewed journal.

Writing Group

Name	Position	Twitter
**Kitty Hiu Fung Wong**	Medical Student, University of Bristol, UK.	@kittywonghf
**David Bosanquet**	Senior Fellow Endovascular Surgery, North Bristol NHS Trust, UK.	@davebosanquet
**Christopher Twine**	Consultant Vascular surgeon, North Bristol NHS Trust, UK.	N/A

In collaboration with VERN (@VascResearchNet).
With support from the Rouleaux Club (@RouleauxClub).

About VERN

Trainee research collaboratives (TRC) have been developed over the last decade to deliver high-quality, multi-centre research. This is achieved by carrying out “snapshot”, protocol-driven, pragmatic audit or research projects undertaken by a regional, national, or international network of trainees during a limited time frame[6]. In promoting multi-centre participation, TRC studies are better statistically powered with greater clinical relevance and generalisability than traditional single-centre research. Trainees are also supported in professional development and academic competencies by participating in TRCs, since all contributing authors are listed as PubMed-citable collaborators under a corporate authorship model, with or without “top-line” authors, in accordance with the National Research Collaborative (NRC) authorship guidelines[5].

The Vascular and Endovascular Research Network (VERN) is a multidisciplinary collaborative founded in 2014 with the goal of undertaking and promoting high-quality vascular audit and research across the UK. Since 2015, VERN is the official UK vascular trainee research collaborative, working in parallel with the UK vascular trainees group, the Rouleaux Club[7]. To date, VERN has delivered UK wide audits of current practice, including an audit of cardiovascular risk management for patients enrolled in abdominal aortic aneurysm surveillance, which has been presented at national vascular meetings and published in the European Journal of Vascular and Endovascular Surgery[8]. Recently, VERN has also published an observational study of the medical management of patients with peripheral artery disease in the British Journal of Surgery[9].

In addition, VERN is currently conducting an international, multi-centre prospective audit on Groin wound Infection after Vascular Exposure (GIVE), engaging 28 centres from 6 countries with nineteen centres further awaiting registration (see www.vascular-research.net).

References

1. National Institute for Health and Care Excellence. Peripheral arterial disease: diagnosis and management. https://www.nice.org.uk/guidance/cg147/chapter/Recommendations#secondary-prevention-of-cardiovascular-disease-in-people-with-peripheral-arterial-disease (2012).
2. Gerhard-Herman, M.D., *et al.* 2016 AHA/ACC Guideline on the Management of Patients With Lower Extremity Peripheral Artery Disease: A Report of the American College of Cardiology/American Heart Association Task Force on Clinical Practice Guidelines. *J Am Coll Cardiol.* 2017;69:e71–e126.
3. Singh, P., *et al.* Peripheral interventions and antiplatelet therapy: Role in current practice. *World J Cardiol*. 2017;9(7):583.
4. Visonà A., *et al*. Antithrombotic treatment before and after peripheral artery percutaneous angioplasty. *Blood Transfus*. 2009;7:18–23.
5. lencowe N, Glasbey J, Heywood N, Kasivisvanathan V, Lee M, Nepogodiev D et al. Recognising contributions to work in research collaboratives: Guidelines for standardising reporting of authorship in collaborative research. International Journal of Surgery. 2018;52:355-360.
6. Bhangu A, Kolias A, Pinkney T, Hall N, Fitzgerald J. Surgical research collaboratives in the UK. The Lancet. 2013;382(9898):1091-1092.
7. Bosanquet D, Stather P, Sidloff D, Dattani N, Shalhoub J, Pancholi J et al. How to Engage in Trainee-led Multicentre Collaborative Vascular Research: The Vascular and Endovascular Research Network (VERN). European Journal of Vascular and Endovascular Surgery. 2016;52(3):392.
8. Saratzis A, Dattani N, Brown A, Shalhoub J, Bosanquet D, Sidloff D et al. Multi-Centre Study on Cardiovascular Risk Management on Patients Undergoing AAA Surveillance. European Journal of Vascular and Endovascular Surgery. 2017;54(1):116-122.
9. Saratzis A, Jaspers N, Gwilym B, Thomas O, Tsui A, Lefroy R et al. Observational study of the medical management of patients with peripheral artery disease. British Journal of Surgery. 2019;106(9):1168-1177.

Appendix A: Survey Summary

These questions pertain to your routine practice or referral practice for percutaneous endovascular intervention in the lower limb for critical ischaemia. This would apply to a patient with no other indication for specific antiplatelet or anticoagulation.

Part 1 – Pre-procedural loading

A pre-op loading is defined as any short-term loading dose of drug prior to lower limb endovascular intervention, including any 'slow loading' a few days before the procedure.

Q1. What is your preferred antiplatelet/anticoagulant agent pre-operatively? (Select all that apply)

Tick	Drug
	Aspirin 75mg
	Clopidogrel 75mg
	Dual antiplatelet therapy (aspirin and clopidogrel)
	Other antiplatelet or dual regime (please specify): ____________________
	Warfarin
	Direct oral anticoagulant (DOAC)
	No preference

Q2. Do you stop antiplatelet/anticoagulant agents pre-procedurally?

Drug	Yes	No
Aspirin 75mg monotherapy		
Clopidogrel 75mg monotherapy		
Dual antiplatelet therapy (aspirin and clopidogrel) to monotherapy		
Warfarin		
Direct oral anticoagulant (DOAC)		

Q3. Which antiplatelets/anticoagulant do you give intra-procedurally? (select all that apply)

- Intra arterial or IV heparin
- Antiplatelet agent (please specify agent and dose)

Part 2 – Post-procedural

A post-procedural drug protocol is defined as long-term drug prescription after peripheral endovascular interventions.

Q1. In general, what do you prescribe post-procedurally for antiplatelet/anticoagulant therapy?
N.B. antithrombotic regimes divided by specific devices and anatomical sites are in subsequent questions.

Tick	Drug
	Aspirin 75mg
	Clopidogrel 75mg
	Dual antiplatelet therapy (aspirin and clopidogrel)
	Other antiplatelet or dual regime (please specify): ____________________
	Warfarin
	Direct oral anticoagulant (DOAC)
	No preference

Q2. What is your preferred antiplatelet / anticoagulant therapy after the following procedures?

	Aspirin	Clopidogrel	Dual antiplatelet therapy	Warfarin	DOAC	Other anticoagulant	N/A
Balloon angioplasty (PTA)							
Covered stent (CS)							
Bare metal stent (BMS)							
Drug eluting Stent (DES)							
Drug coated balloon (DCB)							

Q3. What is your preferred antiplatelet / anticoagulant therapy after angioplasty in the following territories?

	Aspirin	Clopidogrel	Dual antiplatelet therapy	Warfarin	DOAC	Other anticoagulant	N/A
Iliac artery							
Superficial femoral artery (SFA)							
Popliteal artery							
Tibial / crural arteries							

Q4. What influences your antiplatelet/anticoagulant choice?
(Select all that apply)

- Better patency
- Lower risk of amputation
- Lower risk of death
- Success rate of the procedure
- The drug best fits with this type of stent / balloon
- Instructions provided by manufacturer of device
- Improved side effects profile
- Others (please specify): ________________________________

Part 3 – Further research

Q1. Do you believe there is clear evidence to guide your choice of antiplatelet / anticoagulation after peripheral endovascular intervention?

- Yes
- No

Q2. If a randomised controlled trial (RCT) exploring this topic is available to recruit, would you be prepared to randomize into the trial?

- Yes
- No

Any other comments:

